# CRAFTING AN UNDERGRADUATE CONCENTRATION IN AGING ACROSS THE UNIVERSITY AND ASSESSING COMPETENCIES

**DOI:** 10.1093/geroni/igac059.1972

**Published:** 2022-12-20

**Authors:** Kristine Mulhorn

**Affiliations:** Drexel University, Wallingford, Pennsylvania, United States

## Abstract

In the last few years, our Health Administration Department has grown its undergraduate offerings in aging and now is promoting those courses as a concentration available for any major. A recent collaborative effort between our college and the honors college, our university has created a university-wide set of courses across all majors related to topics in aging. In our university, students can pursue a concentration with as few as three courses that are relevant to their majors. Usually, the courses in a concentration are taught by those in the major. However, the global issue of aging can be taught as a specialty area for many majors. To ensure students meet the learning objectives, the following assessment plan was developed for three key courses offered by the Health Administration Department—Healthy Aging, Aging and the Law and either Aging in a Global Context or Aging Services Management. First, a curricular map of the areas of competency within our program are outlined and then the content and areas of assessment are considered based on whether the competencies are “Introduced”, “Reinforced,” and “Mastered.” Next, the key assignments in each course within the concentration were aligned with the Program Objectives for the Health Administration bachelor’s program to assess student achievement of key competencies. In the process of offering the concentration in Aging across multiple majors, it is important for various departments to understand how courses in aging add valuable competencies regardless of their major.

